# Diagnostic Value of Radiomics Analysis in Contrast-Enhanced Spectral Mammography for Identifying Triple-Negative Breast Cancer

**DOI:** 10.3389/fonc.2021.773196

**Published:** 2021-12-23

**Authors:** Yongxia Zhang, Fengjie Liu, Han Zhang, Heng Ma, Jian Sun, Ran Zhang, Lei Song, Hao Shi

**Affiliations:** ^1^ Department of Medical Imaging, Shandong Qianfoshan Hospital, Cheeloo College of Medicine, Shandong University, Jinan, China; ^2^ Department of Radiology, Yantai Yuhuangding Hospital, Yantai, China; ^3^ Marketing Research Department, Huiying Medical Technology Co. Ltd, Beijing, China; ^4^ Department of Geratology, Yantai Yuhuangding Hospital, Yantai, China

**Keywords:** triple-negative breast cancer, radiomics, contrast-enhanced spectral mammography, breast cancer, molecular subtypes

## Abstract

**Purpose:**

To evaluate the value of radiomics analysis in contrast-enhanced spectral mammography (CESM) for the identification of triple-negative breast cancer (TNBC).

**Method:**

CESM images of 367 pathologically confirmed breast cancer patients (training set: 218, testing set: 149) were retrospectively analyzed. Cranial caudal (CC), mediolateral oblique (MLO), and combined models were built on the basis of the features extracted from subtracted images on CC, MLO, and the combination of CC and MLO, respectively, in the tumour region. The performance of the models was evaluated through receiver operating characteristic (ROC) curve analysis, the Hosmer-Lemeshow test, and decision curve analysis (DCA). The areas under ROC curves (AUCs) were compared through the DeLong test.

**Results:**

The combined CC and MLO model had the best AUC and sensitivity of 0.90 (95% confidence interval: 0.85–0.96) and 0.97, respectively. The Hosmer–Lemeshow test yielded a non-significant statistic with *p-value* of 0.59. The clinical usefulness of the combined CC and MLO model was confirmed if the threshold was between 0.02 and 0.81 in the DCA.

**Conclusions:**

Machine learning models based on subtracted images in CESM images were valuable for distinguishing TNBC and NTNBC. The model with the combined CC and MLO features had the best performance compared with models that used CC or MLO features alone.

## Introduction

Triple-negative breast cancer (TNBC) accounts for 10–20% of all diagnosed breast cancers (1). Given the lack of the expression of human epidermal growth factor receptor-2 (HER-2) and estrogen and progesterone receptors, which can be used for targeted therapy, TNBC is difficult to treat and has a high recurrence and metastasis rate, and a low survival rate (2).

Immunohistochemistry, which analyzes part of the tumor tissue obtained by invasive biopsy or surgery, is commonly used for assessing the molecular subtype of breast cancer. However, given the spatial and temporal heterogeneity of breast tumors (3), the accuracy of biopsy is limited. In addition, invasive biopsy is at risk of side effects such as infection, bleeding, and implant metastasis. Therefore, an alternative method is necessary to assess the molecular subtype of the breast cancer completely and non-invasively.

Radiomics is a method of extracting quantitative features from routine medical images (4). These quantitative features, defined as radiomic features, reflect the characteristics of the whole region of interest (ROI) in medical images (5). Several previous studies have explored the value of radiomic features in predicting TNBC based on MRI (6, 7) and mammography (8, 9). However, MRI could not be performed in patients with some medical implants, such as magnetic cardiac pacemakers, defibrillators, and metallic clips. The high cost of MRI also limits its clinical application. Mammography only focuses on morphology, without functional information, which limits its clinical application (10). Moreover, the outline of the tumor is not sharp enough, particularly in dense breast tissue (11).

Contrast-enhanced spectral mammography (CESM) is a novel medical imaging method (12). In CESM, low-energy and subtracted images are obtained using a contrast-enhancing agent at two levels of energy (13). The low-energy image is equal to a standard 2D mammography image, and the subtracted image mostly shows the microcirculation characteristics in the breast in which neovascularization is highlighted (10, 14).

Studies focusing on the value of radiomics analysis based on CESM for the prediction of TNBC are rare. The value of CESM-based radiomics has been preliminarily explored in previous studies (15, 16) to differentiate TNBC from other types of breast cancer. Given the small number of patients, particularly for patients with TNBC, the prediction models in their study were not validated in the testing set. The results in their studies are not highly reliable. Thus, the value of CESM-based radiomics for identifying TNBC should be further explored. In this study, a larger population of patients divided into training and testing sets was used to evaluate the diagnostic value of CESM for identifying TNBC. Furthermore, radiomics models based on radiomic features from cranial caudal (CC) and mediolateral oblique (MLO) views and their combination were built to explore whether the extracting features in different views impact the performance of the prediction models.

## Methods and Materials

### Patients

Patients who underwent CESM between July 2017 and June 2020 were retrospectively analyzed. The inclusion criterion was as follows: (a) patients were pathologically confirmed with breast cancer. The exclusion criteria were as follows: (a) molecular subtype of tumor was not available in the pathological result; (b) tumor not present or not complete in the subtracted image on CC or MLO; (c) excessive glandular overlapped with lesion to influence the segmentation of lesion; (d) underwent treatment prior to CESM; (e) incomplete clinical information, and (f) poor image quality (e.g., remarkable motion and susceptibility artefacts). Patients who underwent CESM between July 2017 and October 2019 were included in the training cohort. A total of 664 patients (109 TNBC and 555 non-TNBC patients) were included in the training set. To resolve the class imbalance problem, 446 non-TNBC patients were randomly excluded. Finally, 109 TNBC and 109 non-TNBC patients were included in the training set. Patients who underwent CESM between November 2019 and June 2020 were included in the testing set. The testing set retained its original distribution of TNBC and non-TNBC patients (30 TNBC and 119 non-TNBC patients). The immunohistochemical results and age of patients were acquired from the electronic medical record system. This retrospective analysis was approved by the local Ethics Committee of our institution, and the requirement for patient informed consent was waived.

### CESM Examination Parameters

CESM was performed using a GE Senographe Essential mammography unit (GE Healthcare, Milwaukee, WI, USA). Iohexol (350 mg I/ml) was injected intravenously at a dose of 1.3 ml/kg and speed of 3.0 ml/s. The CESM examination consisted of a low-energy exposure [kilovolt (peak) of 26–31 kV], immediately followed by a high-energy exposure [kilovolt (peak) of 45–49 kV]. Automatic exposure control (AEC) was used to optimize X-ray parameters automatically. Low-energy images and subtracted images on CC and MLO were acquired in 5 min with a recombination algorithm. No severe adverse events occurred due to contrast administration.

### ROI Segmentation

**Figure 1:**
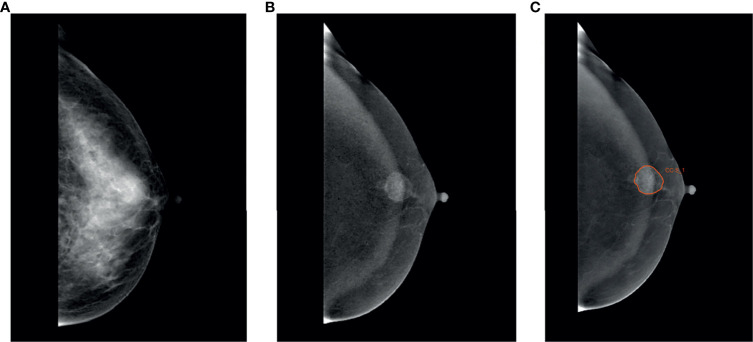
Region of interest was segmented. A 49-year-old woman with TNBC in the left breast. **(A)** Low-energy, craniocaudal view. **(B)** Subtracted image, craniocaudal view. **(C)** TNBC was manually segmented in the subtracted image manually.

The ROI was segmented on CC and MLO by two trained radiologists (Readers 1 and 2, each with 5 years of diagnosis experience in CESM) by using Radcloud (Huiying Medical Technology Co., Ltd, Beijing, China, http://radcloud.cn) in subtracted images (**Figure 1**). ROIs encompassed the entire enhancing lesion. The tumor with the largest diameter was selected for segmentation when the breast cancer was multifocal. The segmentation work on 70 randomly selected patients was first performed by Readers 1 and 2 simultaneously. Reader 1 repeated the segmentation work 2 weeks later. The segmentation work on the remaining 297 patients was finished by Reader 1. Readers 1 and 2 were blind to the results of the pathological examination. An experienced radiologist (Reader 3, with 13 years of diagnosis experience in breast medical images) supervised segmentation work. Revision will be applied if necessary (e.g., Readers 1 and 2 selected different tumors on the same patient, or contour of lesion was not drawn precisely).

### Radiomics Feature Extraction

For each CESM sequence on each image, 1,409 radiomics features were extracted using a tool from the Radcloud platform, which extracted radiomics features from medical image data with a large panel of engineered hard-coded feature algorithms (http://mics.radcloud.cn/#/project). The 1,409 features obtained were divided into four main categories: first-order statistics, shape, texture [gray-level co-occurrence (GLCM), gray-level run length (GLRLM), gray-level size zone (GLSZM), neighboring gray tone difference (NGTDM), gray-level dependence (GLDM), Matrices], and higher-order statistics (Laplacian of Gaussian, wavelet, square, square root, logarithm, exponential, gradient, and local binary pattern filters) features. The CC and the MLO feature datasets were merged into the combined dataset.

### Inter- and Intra-Agreement of Radiomics Features

The inter- and intra- agreements of radiomics features were evaluated by using intraclass correlation coefficient (ICC) analysis based on radiomics features for the 70 patients mentioned above. The inter- and intra-ICCs for each radiomic feature were acquired *via* the radiomic features extracted from ROIs segmented by Readers 1 and 2 simultaneously and by Reader 1 at different times. Radiomic features with inter- and intra-ICCs >0.75 were selected for the subsequent statistical analysis.

### Radiomic Features Selection and Radiomic Model Building

Immunohistochemical results were selected as the gold reference. Normalization was applied to rescale all features from the original range to a new range of 0 and 1. Radiomic features in the training set that were not significantly different between patients with and without TNBC were filtered from the CC, MLO, and combined CC and MLO feature datasets by using univariate analysis. After the above filtration, the least absolute shrinkage and selection operator (LASSO) (17) method was used to decrease the high degree of redundancy of radiomic features. The optimal coefficient of regularization (*α*) used for the LASSO method was selected using the inner 10-fold cross-validation in the training set with a maximum iteration of 5,000 *via* the binomial deviance. Subsequently, the radiomic parameters with non-zero coefficients in the LASSO model generated by the entire training set with the optimal α were selected. CC, MLO, and combined radiomic models were built on the basis of the coefficients of each selected feature *via* the LASSO method.

### Evaluation of Radiomic Model

**Figure 2:**
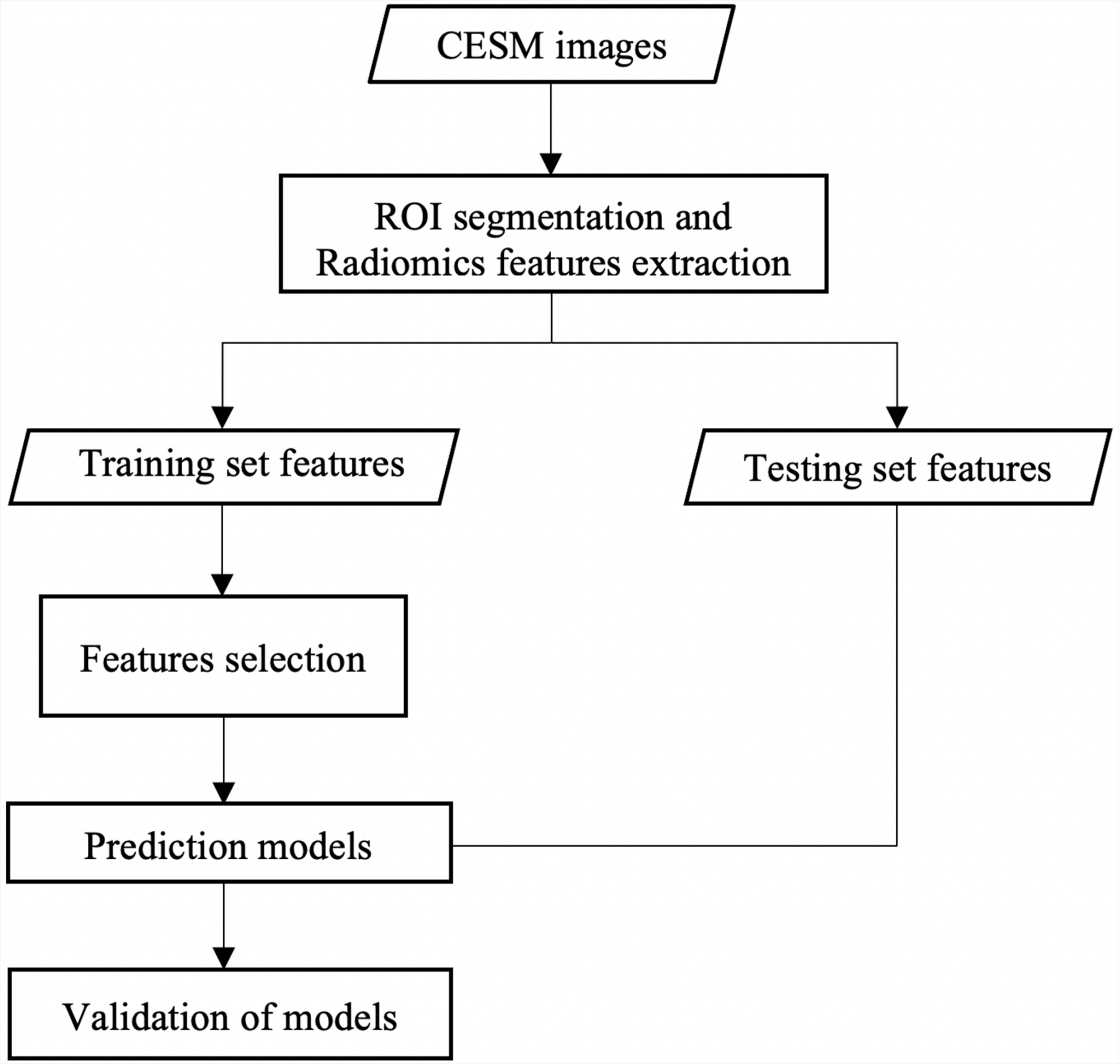
Workflow of this study.

The probabilities of TNBC for patients were acquired through the CC, MLO, and combined models. Respective Youden indexes were calculated and were selected as threshold. If probability was higher than threshold, the respective patient was predicted as TNBC patient. The discrimination ability of the CC, MLO, and combined radiomic models at all thresholds in the training and testing sets was shown through receiver operating characteristic (ROC) curve analysis. The 95% confidence interval (CI) of the area under the ROC curve (AUC) was acquired on the basis of the bootstrapping. The AUCs in the testing set for each model were compared. Prediction models were also evaluated by using the Hosmer–Lemeshow test, which assessed whether the observed event rates matched the expected event rates in the subgroups of the model population. The clinical usefulness of radiomic models in the testing set was evaluated using decision curve analysis (DCA) (18) in the testing set. The DCA measured the net benefit which placed benefits and harms on the same scale at each possible threshold probability. The workflow of this study is presented in **Figure 2**.

### Statistics Analysis

The R (version 3.6.3) was used for statistical analysis. Age and tumor diameter of patients with and without TNBC in the training and testing sets were statistically analyzed using *t* test or Mann–Whitney U test. The percentage of postmenopausal patients in the training or testing set was statistically analyzed using Chi-Squared Test. Univariate analysis was performed using one-way ANOVA (19) or the Mann–Whitney U test (20). The areas under ROC curves (AUCs) of models in the testing cohort were compared using the Delong test (21). The sensitivities and specificities of models in the testing set were compared based on bootstrap. The reported statistically significant levels were all two-sided, and the statistical significance was set at 0.05.

## Results

### Patients

Among the 218 patients in the training set (age: mean ± SD = 54.57 ± 10.31 years, range = 29–76 years), 109 had TNBC. Of the 149 patients in the testing set (age: mean ± SD = 55.07 ± 9.70 years, range = 27–76 years), 30 had TNBC. The clinical characteristics between TNBC and non-TNBC patients in training and testing sets were not statistically different (*p*>0.05). The characteristics of the patients in the training and testing sets are presented in **Table 1**.

**Table 1:** Characteristics of patients in the training and testing sets.

	Training set		Testing set	
	Characteristics	*p*	Characteristics	*p*
Age, mean ± SD, years	54.57 ± 10.31	0.37	55.07 ± 9.70	0.45
range, years	29–76		27–76	
Postmenopausal patients, no. (%)	130 (60)	0.24	87 (59)	0.29
Tumor diameter, mean ± SD, cm	3.57 ± 2.10	0.39	3.17± 1.97	0.80
range, cm	0.97–10.78		0.58–10.62	
TNBC, No. (%)	109 (50)	–	30 (20)	–
All, no.	218		149	

SD, standard deviation; TNBC, triple-negative breast cancer.

p Values indicated difference in clinical characteristics between TNBC and non-TNBC patients in the training or testing sets.

### Feature Selection and Prediction Model Building

A total of 2,072 radiomics features were discarded for low intra- or inter-class correlations. After univariate analysis, 164, 148, and 312 radiomics features were significantly different (*p* < 0.05) between patients with and without TNBC in the CC, MLO, and combined feature datasets, respectively. A total of 5, 8, and 8 radiomics features (2 from the CC feature dataset and 6 from the MLO feature dataset) were selected as useful radiomics features by the LASSO method in the CC, MLO, and combined feature datasets, respectively. All selected radiomic features were texture features (GLRLM), including that after filter transformation (logarithm in the CC, logarithm and wavelet in the MLO). Two of the 8 radiomics features in the combined model were original GLRLM from the CC; the other 6 features were from the MLO feature datasets. Therefore, the radiomics features in the combined model were the same as part of radiomics features in the CC and MLO models. The selected features in the CC, MLO, and combined prediction models are presented in **Table 2**.

**Table 2:** Features in the CC, MLO, and combined models.

Model	Feature
CC	original_glrlm_ShortRunLowGrayLevelEmphasis_CC
	original_glrlm_ShortRunHighGrayLevelEmphasis_CC
	original_glrlm_ShortRunEmphasis_CC
	logarithm_glrlm_ShortRunLowGrayLevelEmphasis.1_CC
	logarithm_glrlm_ShortRunHighGrayLevelEmphasis.1_CC
MLO	original_glrlm_ShortRunLowGrayLevelEmphasis_MLO
	original_glrlm_ShortRunHighGrayLevelEmphasis_MLO
	original_glrlm_ShortRunEmphasis_MLO
	logarithm_glrlm_ShortRunLowGrayLevelEmphasis.1_MLO
	logarithm_glrlm_ShortRunHighGrayLevelEmphasis.1_MLO
	wavelet.HHH_glszm_ZoneEntropy.12_MLO
	wavelet.LLL_glrlm_ShortRunLowGrayLevelEmphasis.14_MLO
	wavelet.LLL_glrlm_ShortRunHighGrayLevelEmphasis.14_MLO
combined	original_glrlm_ShortRunLowGrayLevelEmphasis_CC
	original_glrlm_ShortRunHighGrayLevelEmphasis_CC
	original_glrlm_ShortRunLowGrayLevelEmphasis_MLO
	original_glrlm_ShortRunHighGrayLevelEmphasis_MLO
	original_glrlm_ShortRunEmphasis_MLO
	logarithm_glrlm_ShortRunLowGrayLevelEmphasis.1_MLO
	logarithm_glrlm_ShortRunHighGrayLevelEmphasis.1_MLO
	wavelet.HHH_glszm_ZoneEntropy.12_MLO

### Validation of Models in the Training and Testing Sets

**Figure 3:**
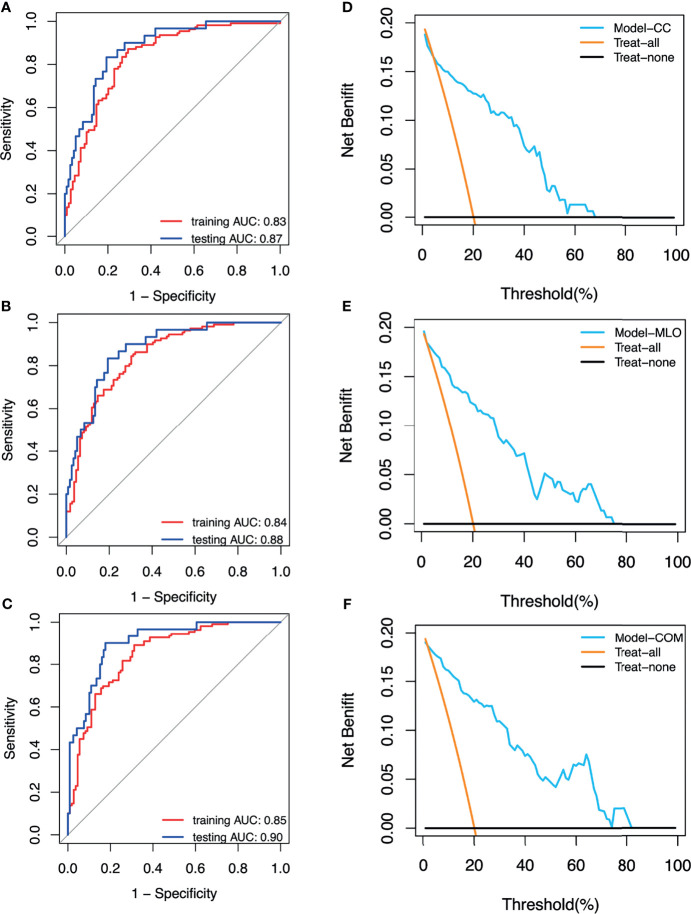
**(A)** Receiver operating characteristic (ROC) curves of the CC model. **(B)** ROC curves of the MLO model. **(C)** ROC curves of the combined model. **(D)** Decision curve of the CC model. **(E)** Decision curve of the MLO model. **(F)** Decision curve of the combined (COM) model.

The AUCs of the CC, MLO, and combined models were 0.87 (95% CI = 0.79–0.95), 0.88 (95% CI = 0.81–0.94), and 0.90 (95% CI = 0.85–0.96), respectively, in the testing set. The AUC of the combined model was higher than that of the CC (*p* > 0.05) and MLO (*p* > 0.05) models in the testing set. The combined model also reached the highest sensitivity (0.97) compared with the CC (0.93, *p* > 0.05) and MLO (0.93, *p* > 0.05) models in the testing set. The AUCs of the CC, MLO, and combined models were 0.83 (95% CI = 0.78–0.89), 0.84 (95% CI = 0.79–0.89), and 0.85 (95% CI = 0.80–0.90), respectively, in the training set. The sensitivity values of the CC, MLO, and combined models in the training set were 0.87, 0.84, and 0.89, respectively. In addition, the specificity values of the CC, MLO, and combined CC and MLO models were 0.60, 0.59, and 0.69, respectively, in the testing set and 0.71, 0.69, and 0.55, respectively, in the training set. The specificity of the combined model was statistically higher than that of the CC and MLO models (*p* < 0.05). The Hosmer–Lemeshow test yielded non-significant statistical difference with *p* = 0.28, 0.46 and 0.59 for the CC, MLO, and combined models, respectively. AUCs, sensitivities, and specificities of CC, MLO, and combined models are shown in **Table 3**. All three models were clinically useful in DCA. If the threshold was between 0.05 and 0.67, the CC model added more net benefit than the “treat-all” and “treat-none” models. If the threshold was between 0.03 and 0.74, the MLO model added more net benefit than the “treat-all” and “treat-none” models. If the threshold was between 0.02 and 0.81, the combined model added more net benefit than the “treat-all” and “treat-none” models. The ROC and decision curves are shown in **Figure 3**.

**Table 3:** Validation of models in the training and testing sets.

	Training set			Testing set		
	AUC	Sensitivity	Specificity	AUC	Sensitivity	Specificity
CC	0.83	0.87	0.71	0.87 (CC *vs* combined, *p* > 0.05)	0.93 (CC *vs* combined, *p* > 0.05)	0.60 (CC *vs* combined, *p* < 0.05)
MLO	0.84	0.84	0.69	0.88 (MLO *vs* combined, *p* > 0.05)	0.93 (MLO *vs* combined, *p* > 0.05)	0.59 (MLO *vs* combined, *p* < 0.05)
Combined	0.85	0.89	0.55	0.90	0.97	0.69

## Discussion

This study indicated that machine learning models based on the subtracted images in CESM images were valuable for distinguishing TNBC and non-TNBC, and such models showed good performance. The combined model based on the combination of CC and MLO features had the best performance with the highest AUC, sensitivity, and specificity in the testing set. The best performance of the combined model compared with the CC and the MLO models may be explained by the combination of the CC and MLO radiomic feature sets, which contained more information than the CC or MLO radiomic feature set alone.

In the previous CESM study of MARINO et al. (16), only100 patients (12 patients with TNBC) were included. All 12 patients with TNBC were correctly predicted. The performance of the model in that study seemed to be better than that of our study. However, the small number of patients, the imbalance of class, and the lack of a testing set affected the robustness of the results. LA FORGIA D et al. (15) evaluated CESM-based radiomic features to predict TNBC. A total of 52 patients (68 breast cancers) were included. The obtained AUC of 76.80% was lower than the AUC obtained in our study. The results in that study were also not reliable enough. In addition, the number of patients (367) included in our study was larger than that included in the abovementioned previous studies. The number of patients with TNBC and non-TNBC in the training set was equal to reduce the effect of class imbalance (22).

Radiomics features serve as the bridge between medical images and machine learning. In our study, 1,409 radiomics features, including first-order statistics, shape, texture (GLCM, GLRLM, GLSZM, NGTDM, and GLDM), and high-order statistics (Laplacian of Gaussian, wavelet, square, square root, logarithm, exponential, gradient, and local binary pattern filters), were included. The features are more comprehensive than those in the study of MARINO et al. (16) (300 features) and LA FORGIA D et al. (15) (7 features). Our study thoroughly explored the value of CESM radiomics features to predict TNBC. All radiomics features included in the CC, MLO, and combined models were texture features, including that after filter transformation. Texture features quantify the inter-voxel relationships in an image. Such features describe microscopic characteristics in CESM images. Texture features can capture the unique aspects of the biological heterogeneity of breast cancers and contain part of pathological characteristics related to TNBC.

The value of MRI-based radiomics has been explored in previous studies (6, 7, 23) to differentiate TNBC from others. In the study of WANG et al. (6), the model based on radiomic features in the tumor region of dynamic contrast-enhanced MRI (DCE-MRI) has achieved an AUC of 0.78 in predicting TNBC. Although the specificity of this model (0.95) was higher than that of the combined model in our study (0.69), the sensitivity of the combined model in our study (0.97) was higher (0.33). In addition, Leithner et al. (23) evaluated the performance of radiomics features from DCE-MRI and the apparent diffusion coefficient (ADC) to the assess the breast cancer molecular subtype and yielded an AUC of 0.86 for predicting TNBC. In our study, the combined CC and MLO model reached an AUC of 0.90. Moreover, patients with TNBC in our study were much more than those studies, which can comprehensively represent the characteristics of TNBC. This result shows that the ability of CESM-based radiomic features is not worse than that of MRI to predict TNBC.

The value of radiomics features based on mammography in predicting TNBC has also been explored. MA et al. (9) investigated the association of radiomic features extracted from mammogram images with molecular subtypes of breast cancer and yielded an AUC of 0.87 for TNBC *vs.* non-TNBC. The AUC of this study was slightly lower than that of our study. The model with combined CC and MLO radiomic features has achieved better performance compared with that with CC or MLO radiomic features alone. This result is consistent with that of our study. However, the AUC of the CC-view-based model (0.695) was lower than that of MLO-view- and CC-and-MLO-view- based models (0.853 and 0.865, respectively) in their study. In our study, the CC-view-based model and MLO-view-based model also performed well, of which AUCs were just slightly lower than that of the combined model. Therefore, we thought that if complete images both of CC and MLO could not be obtained, then CESM features extracted from a single orientation can also be used to identify TNBC. The present study has several limitations. Firstly, this study was a retrospective and single-center study. Prospective and multicenter studies are needed to verify the results. Secondly, manual segmentation, which is time-consuming and subjective, was applied in this study because the automatic segmentation algorithm is not mature enough. Automatic segmentation algorithms need further development. In addition, as a pilot study, radiomic features extracted from low-energy images were not analyzed. The value of radiomic features extracted from low-energy images will be explored in future studies. What is more, specificities of models were low. Parameter optimization methods and more model algorithms will be applied in future studies to achieve good performance. Finally, no clinical factor was used to build the prediction model. Further studies are needed to develop and validate the prediction model incorporating radiomic features and clinical factors.

## Conclusion

In conclusion, the radiomic features extracted from subtracted images in the CESM were valuable to the identification of TNBC. The prediction model based on the combination of CC and MLO features had the best performance. Better prediction models incorporating radiomic features extracted from low-energy, subtracted images and clinical factors are expected to be developed and validated in future works.

## Data Availability Statement

The original contributions presented in the study are included in the article/supplementary material. Further inquiries can be directed to the corresponding authors.

## Ethics Statement

The studies involving human participants were reviewed and approved by Ethics Committee of the Yantai Yuhuangding Hospital. Written informed consent for participation was not required for this study in accordance with the national legislation and the institutional requirements.

## Author Contributions

YZ, HM, LS, and HS designed the study. YZ, HZ, JS, and FL collected data. YZ, HZ, and RZ processed data. YZ, FL, and LS drafted the manuscript. All authors contributed to the article and approved the submitted version.

## Conflict of Interest

Author RZ was employed by company Huiying Medical Technology Co. Ltd.

The remaining authors declare that the research was conducted in the absence of any commercial or financial relationships that could be construed as a potential conflict of interest.

## Publisher’s Note

All claims expressed in this article are solely those of the authors and do not necessarily represent those of their affiliated organizations, or those of the publisher, the editors and the reviewers. Any product that may be evaluated in this article, or claim that may be made by its manufacturer, is not guaranteed or endorsed by the publisher.
